# Supplementary material for: A microfluidic-induced C. elegans sleep state
Source: Nat Commun. 2019 Nov 6;10:5035. doi: 10.1038/s41467-019-13008-5 (PMC6834590; doi:10.1038/s41467-019-13008-5)
Supplement: Supplementary file 1 — Supplementary Information [file 41467_2019_13008_MOESM1_ESM.pdf]

Title:

A Microfluidic-Induced *C. elegans* Sleep State

Authors:

Daniel L. Gonzales[1,2], Jasmine Zhou[3], Bo Fan[2], Jacob T. Robinson[1-4*]

Affiliations:

[1] Applied Physics Program, Rice University, 6100 Main St., Houston, TX 77005, USA.

[2] Department of Electrical and Computer Engineering, Rice University, 6100 Main St., Houston, TX 77005, USA.

[3] Department of Bioengineering, Rice University, 6100 Main St., Houston, TX 77005, USA.

[4] Department of Neuroscience, Baylor College of Medicine, One Baylor Plaza, Houston, TX 77030, USA.

*Correspondence to: jtrobinson@rice.edu.

Supplemental Information:

Supplementary Figs. 1 to 8

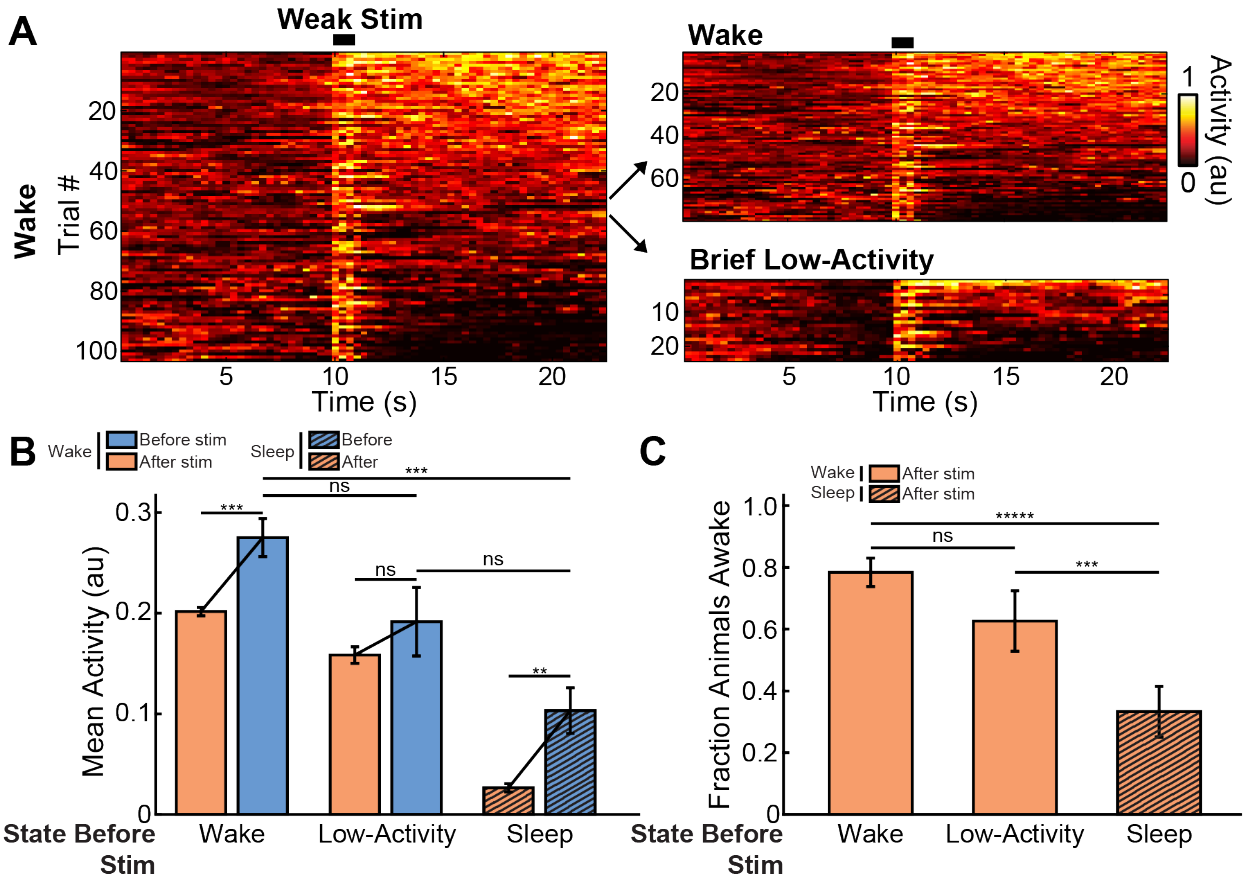

Supplementary Figure 1. Further comparison of behavioral responses to weak mechanical stimulation. *(A) (Left)* Heatmap of all behavioral activity from all wake animals receiving a weak mechanical stimulus (n = 176 total trials, note that in Figure 2 only the first 25 animals are shown for clarity). *(Right)* We further parsed this data set into "Wake" and "Low-Activity" states, where Low-Activity is defined as having an average behavioral activity level below the sleep threshold for a short 3 s period immediately before the stimulus. *(B)* Average behavioral activity before and after the weak stimulus for animals in the Wake and Low-Activity states prior to the stimulus. We also compared these results to animals in the Sleep state prior to the stimulus (same data as Figure 2D, error bars are sem, ***p < 0.001, Kruskal-Wallis with a *post-hoc* Dunn-Sidak test). *(C)* Fraction of animals awake following the stimulus for the Wake and Low-Activity states. We also compared these data to the Sleep state (same data as Figure 2E). Animals in the Sleep state are less likely to respond to weak stimuli and transition to wakefulness, compared to animals in the wake state regardless of their activity level prior to the stimulus. (Error bars are standard deviation, calculated by bootstrapping each data set with 5000 iterations; ns = not significant, ***p<0.001, ****p<0.0001; significance was calculated by data resampling 5000 iterations and a *post hoc* Bonferroni correction).

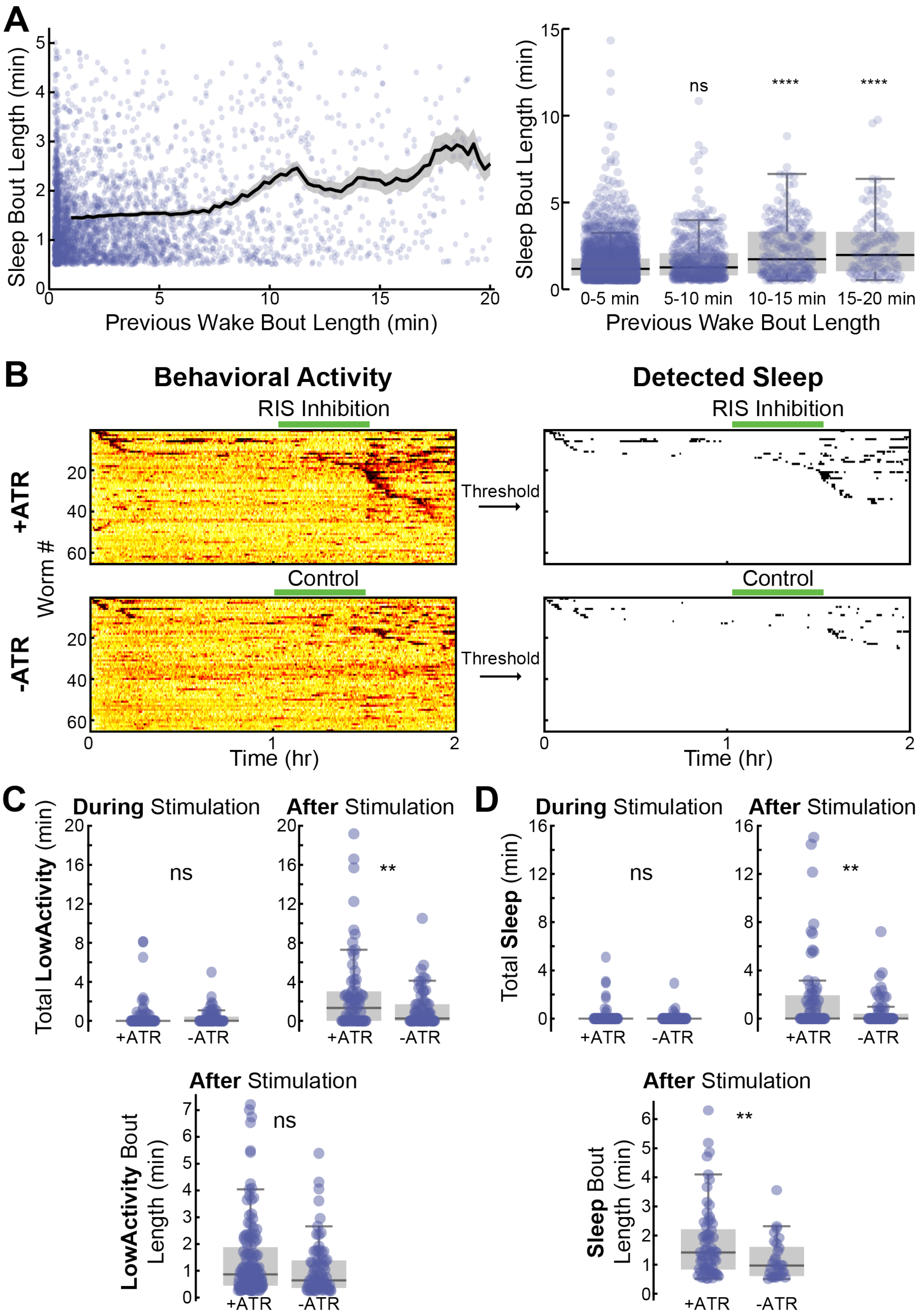

Supplementary Figure 2. Evidence for homeostasis in microfluidic-induced sleep. *(A)* Sleep bout length is dependent on the length of the previous wake bout. *(Left)* Data points represent individual sleep and wake bout pairs from data collected from 50 and 500 μm chambers with WT animals

(see Figure 1, 4 hr-long recordings and Figure 6, 2 hr-long recordings). *(Right)* Wake bout lengths binned into 5 min windows. As the wake bout length increases, the following sleep bouts increase in length, suggesting a micro-homeostatic mechanism (ns = not significant, ****p<0.0001 compared to 0-5 min data set, Kruskal-Wallis with a *post-hoc* Dunn-Sidak test). *(B-D)* Animals showed a homeostatic rebound in response to prolonged RIS optogenetic inhibition. *(B) (Left)* Heatmaps of animal behavioral activity during an optogenetic assay where green light illumination was delivered between 1-1.5 hr. *(Right)* Raster plots of detected sleep bouts. +ATR animals were grown with OP50 seeded with *all-trans retinal*. -ATR animals were not, thus making optogenetic inhibition ineffective. *(C)* We used a threshold 66% higher than the sleep threshold to detect all low-activity bouts (these include sleep and other low-activity behaviors). +ATR animals showed similar behavioral activity during the stimulus, but more low-activity after the stimulus, suggesting that prolonged RIS inhibition led to a homeostatic response following optogenetic inhibition of the sleep-promoting RIS neuron. *(D)* Using our standard threshold for sleep detection, +ATR and -ATR animals surprisingly showed no significant difference in total sleep during illumination. However, +ATR animals showed significantly more sleep following stimulation, suggesting a homeostatic response to prolonged RIS inhibition. In addition, +ATR animals showed longer sleep bouts following illumination. These results suggest that *C. elegans* show increased microfluidic-induced sleep and low-activity following extended RIS inhibition. (ns = not significant, *p<0.05, **p<0.01, unpaired two-sided t-test).

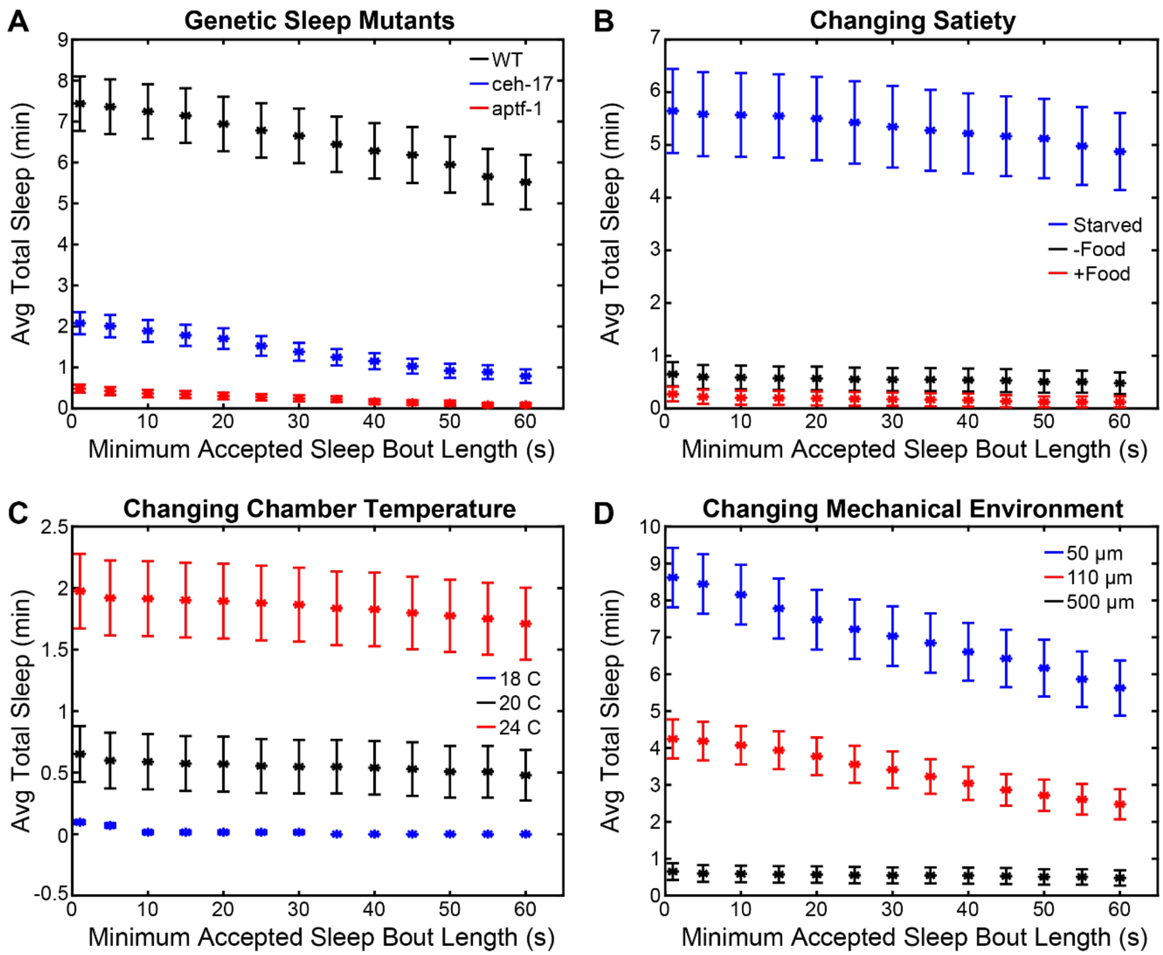

Supplementary Figure 3. Reported sleep trends are unaltered by the choice of minimum sleep time. For all data reported, low-activity bouts lasting less than 30 s were removed in order to reduce false-positive detections (see Methods). Here, we show that reducing this value to even 1 s does not change the reported conclusions. Data sets analyzed here are from various portions of the manuscript. *(A)* Genetic mutant data reported in Figure 2 (chamber widths are 50 µm). *(B)* Satiety data from Figure 6 (chamber widths are 500 µm). *(C)* Temperature data for WT animals in Figure 6 (chamber widths are 500 µm). (*D)* Data from WT animals in which chamber width was changed in Figure 6 (chamber widths vary and are indicated in the legend). As expected, data from large chambers (500 µm width) is highly stable due an easy distinguishing between sleep and wakefulness. More variation is seen in smaller chambers (50 µm width), where animal movement is heavily restricted, and animals exhibit more low-activity bouts that are not necessarily sleep behavior. In all cases however, the reported trends in throughout the manuscript are sound.

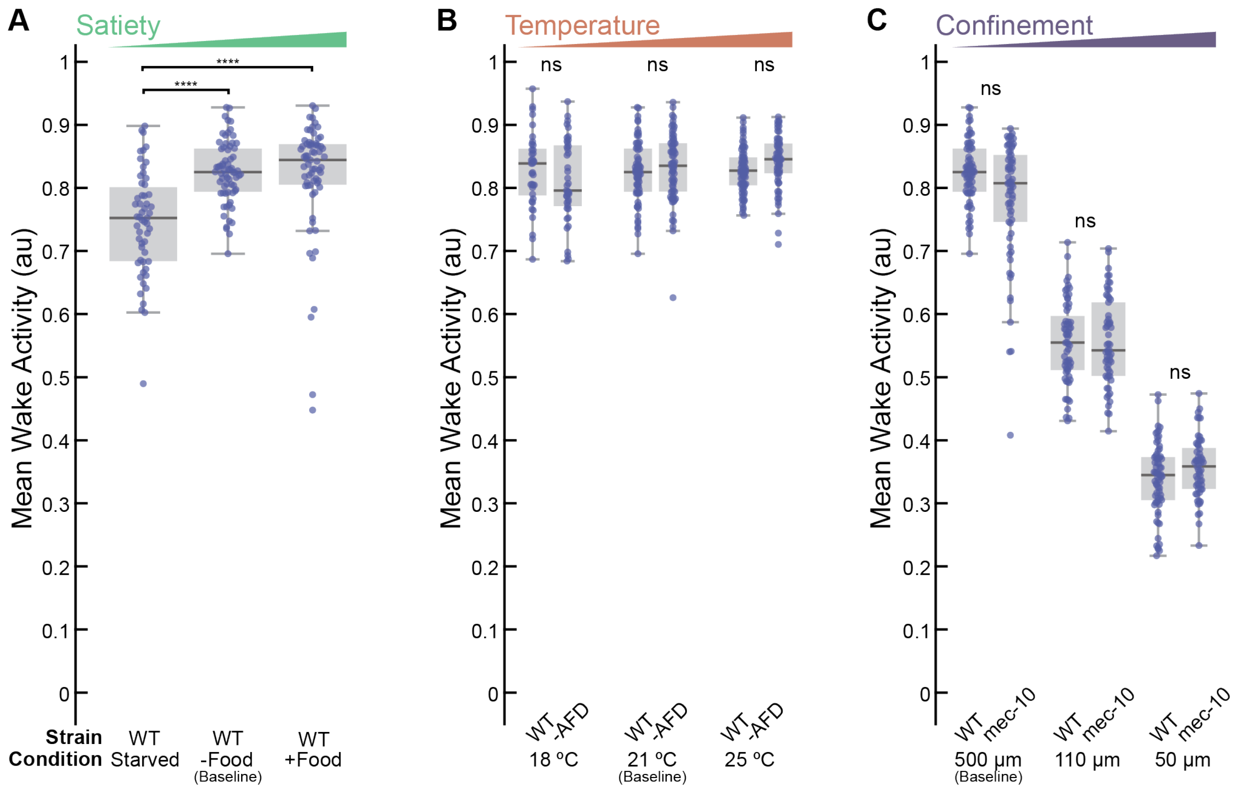

Supplementary Figure 4. Behavioral activity during wakefulness in different environmental conditions. Data is the averaged wake-state behavioral activity for each animal in Figure 6. *(A)* Only WT animals in the starved condition only show less activity compared to -Food and +Food. *(B)* No significant differences were found in either WT or *gcy-23(nj37);gcy-8(oy44);gcy-18(nj38)* mutants (labeled as -AFD) at any microfluidic device temperature. *(C)* As expected, restricting animal movement significantly changes behavioral activity during wakefulness. However, no changes were observed between WT and *mec-10(tm1552)* animals. (****p<0.0001, ns = not significant, Kruskal-Wallis with a *post-hoc* Dunn-Sidak test).

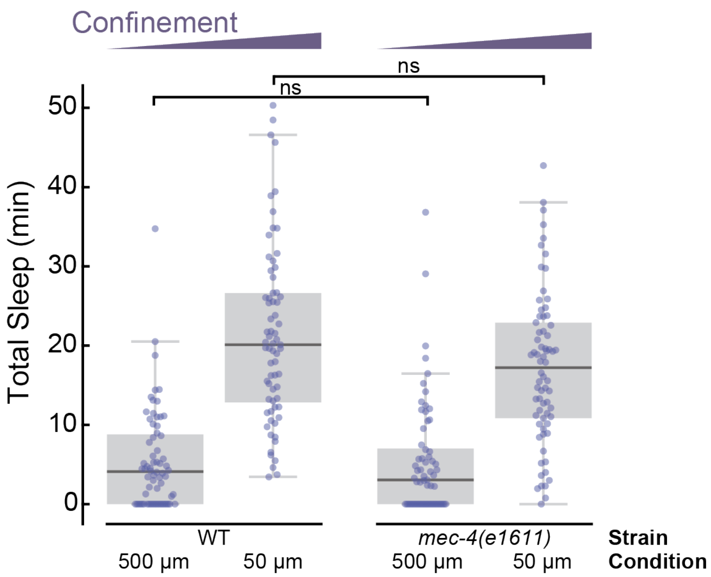

Supplementary Figure 5. *mec-4(e1611)* gentle-touch-defective mutants show same confinement phenotype as WT. When animals are allowed to swim in large 500 µm-wide chambers, mutant and WT strains show the same amount of total sleep. When partially immobilized in 50 µm-wide chambers, each strain shows an increase in total microfluidic-induced sleep, but the phenotypes remain the same. These results indicate that harsh-touch mechanosensory pathways, such as *mec-10(tm1552)* (Figure 6), drive microfluidic-induced sleep. (WT 500 µm n = 68, WT 50 µm n = 69, *mec-4(e1611)* 500 µm n = 65, *mec-4(e1611)* 500 µm n = 70; ns = not significant, Kruskal-Wallis with a *post-hoc* Dunn-Sidak test).

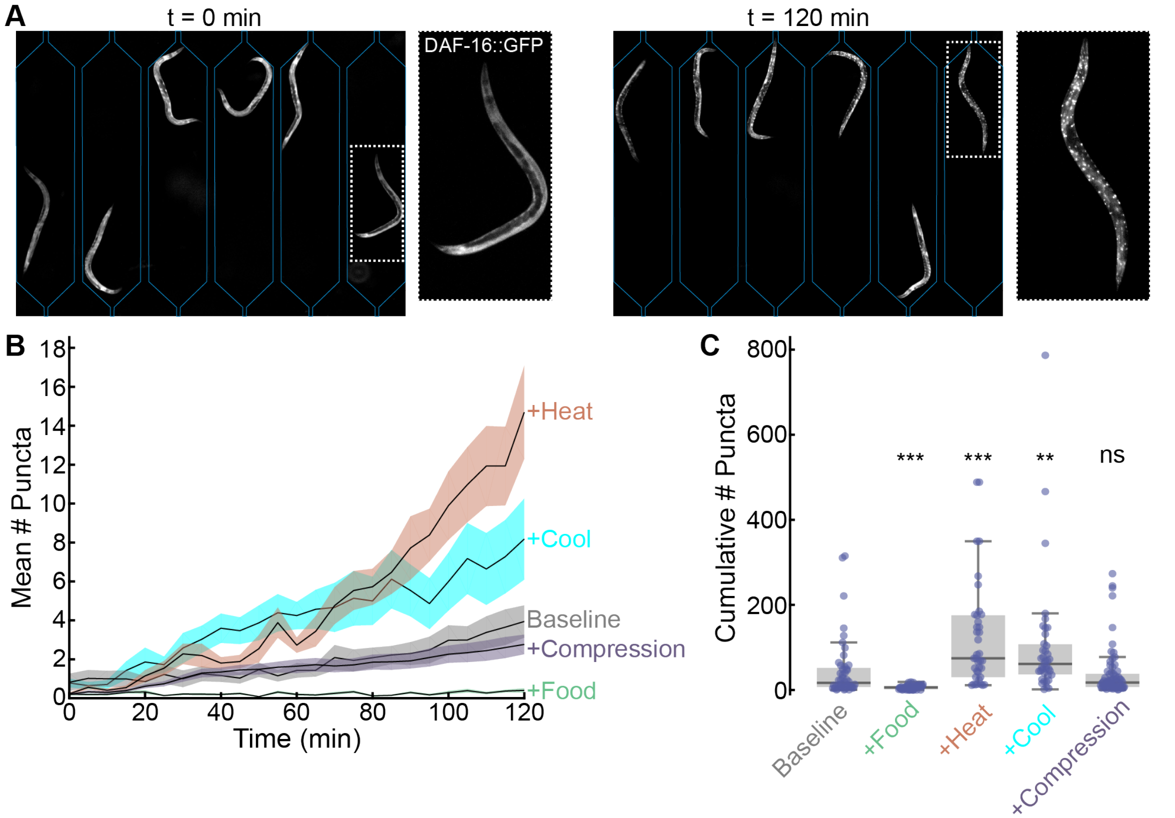

Supplementary Figure 6. DAF-16::GFP imaging shows that microfluidic-induced sleep partially correlates with *C. elegans* stress. *(A)* Fluorescent micrographs (with background subtracted) of DAF16::GFP animals confined to 500 μm-wide chambers during the +Heat condition. Microfluidic chambers are outlined. Left image (t = 0 min) shows diffuse DAF-16::GFP. Over the course of 2 hr, DAF-16 localizes to the nucleus (right image). *(B)* Puncta formation with respect to time for Baseline, +Food, +Heat, +Cool, and +Compression conditions (see Methods, Figure 6). *(C)* Cumulative number of puncta during imaging. Individual data points represent individual animals. (Baseline n = 48, +Food n = 46, +Heat n = 38, +Cool n = 39, +Compression n = 71; **p < 0.01, ***p < 0.001, ns = not significant, Kruskal-Wallis with a *post-hoc* Dunn-Sidak test).

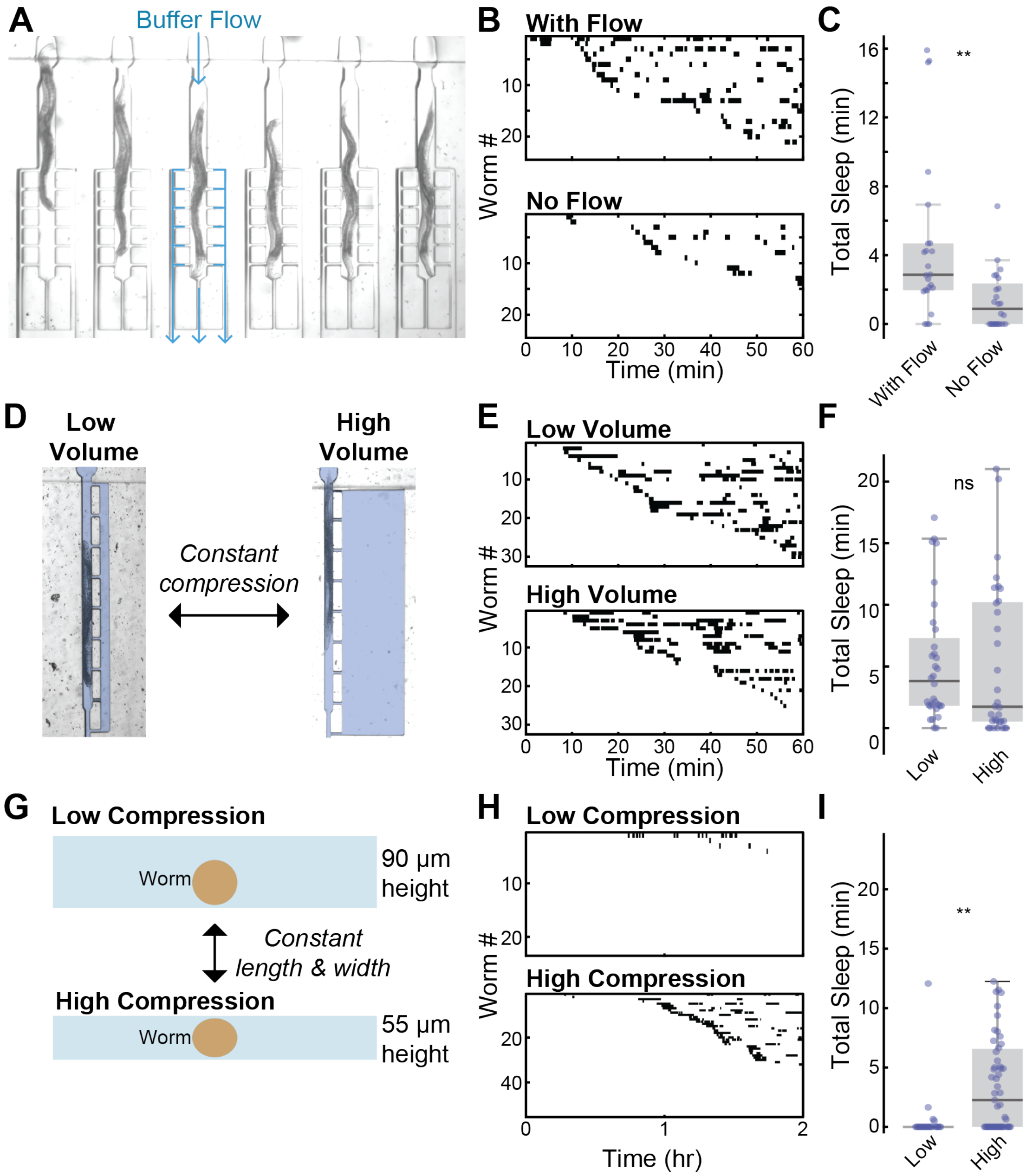

Supplementary Figure 7. Waste buildup, chamber volume, and stress from loading do not strongly influence microfluidic-induced sleep. *(A)* Image of animals confined in microfluidic chambers designed for constantly flowing buffer to stabilize O2 concentrations and remove the buildup of CO2 and other byproducts. Blue paths indicate the direction of flow for a single chamber. Flow rate was ~1 mL/hr. *(B)* Raster plots of detected sleep with and without flow using the geometry in (A). *(C)* We surprisingly observed more sleep with the buffer flow, indicating microfluidic-induced sleep is likely not driven by changing gas concentration levels biological byproducts (n = 24 for each condition). *(D)* Chamber designs for maintaining a constant animal compression while changing fluidic volume. Fluid is false-colored in pale blue. *(E)* Raster plots of detected sleep for each chamber type. *(F)* Animals in chambers of different volume do not show different amounts of sleep (n = 32 for each condition). *(G)* Schematic of chamber cross section that have same width

and length, but different chamber heights. *(H)* Raster plots of detected sleep. *(I)* Sleep in the 90um tall chambers is essentially abolished, demonstrating that the animal loading process and microfluidic environment alone do not drive sleep (n = 22). Sleep dramatically increases in the 55 um tall chambers (n = 50), again demonstrating that the mechanical environment regulates sleep strongly. (ns = not significant, **p < 0.01, unpaired two-sided t-test).

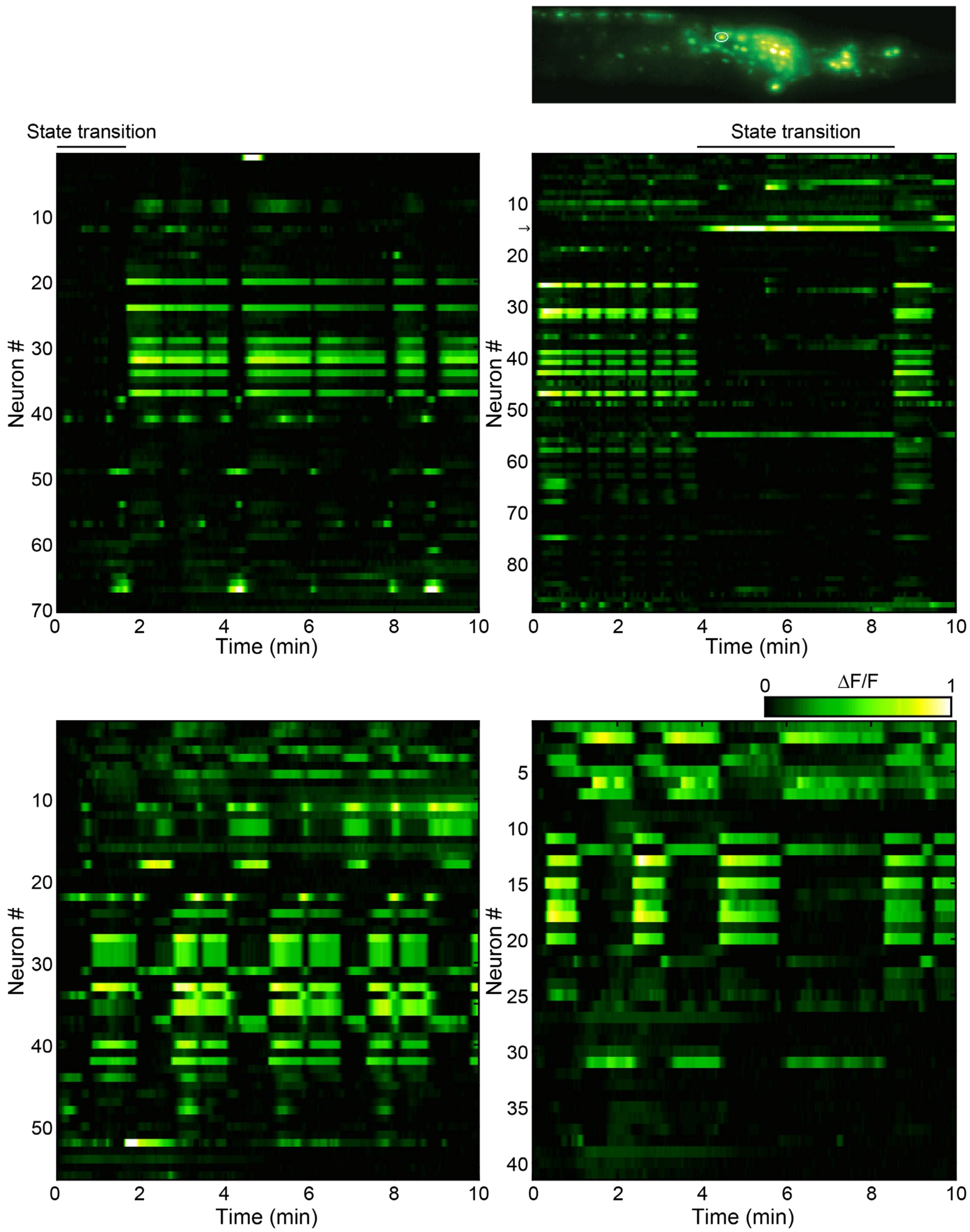

Supplementary Figure 8. Whole-brain imaging in paralyzed animals. Representative volumetric imaging data from four paralyzed animals during whole-brain imaging. The majority of the data resembles previous imaging work during wakefulness [1], wherein many neurons show correlated calcium dynamics. Potential sleep states (top two panels) identified by a global state transition and downregulated neural activity [2,3], are labeled but cannot be confirmed without behavioral readouts. In the top right heatmap, an indicated neuron that increases in activity during potential sleep corresponds to the approximate RIS location (circled neuron in top micrograph). These data

demonstrate that microfluidic-induced sleep can be used as a model behavior for understanding how brain-wide neural circuits drive spontaneous brain state transitions.

Supplementary References:

1. Kato, S. *et al.* Global Brain Dynamics Embed the Motor Command Sequence of Caenorhabditis elegans. *Cell* 163, 656–669 (2015).

2. Nichols, A. L. A., Eichler, T., Latham, R. & Zimmer, M. A global brain state underlies C. elegans sleep behavior. *Science (80-.).* 356, eaam6851:1-9 (2017).

3. Skora, S., Mende, F. & Zimmer, M. Energy Scarcity Promotes a Brain-wide Sleep State Modulated by Insulin Signaling in C. elegans. *Cell Rep.* 22, 953–966 (2018).
