# Supplementary material for: A microfluidic-induced C. elegans sleep state
Source: Nat Commun. 2019 Nov 6;10:5035. doi: 10.1038/s41467-019-13008-5 (PMC6834590; doi:10.1038/s41467-019-13008-5)
Supplement: Supplementary file 3 — Description of Additional Supplementary Files [file 41467_2019_13008_MOESM3_ESM.pdf]

Description of Additional Supplementary Files

File Name: Supplementary Movie 1
Description: Microfluidic-induced sleep in a large microfluidic chamber. (Top) Animal activity calculated by subtracting consecutive frames (see Methods). (Bottom) Video (with background subtracted) of the animal swimming in large microfluidic chamber. The animal is initially in the active state, then spontaneously transitions in and out of quiescence.

File Name: Supplementary Movie 2
Description: Microfluidic-induced sleep in a small microfluidic chamber. (Top) Animal activity trace calculated by subtracting consecutive frames (see Methods). (Bottom) Video (with background subtracted) of the animal partially immobilized in a small microfluidic chamber. The animal is initially in the active state, then spontaneously transitions in and out of quiescence.

File Name: Supplementary Movie 3
Description: Microfluidic-induced sleep is reversible with strong blue light illumination. (Top) Animal activity trace. Blue bar indicates a 5 s light stimulation. (Bottom) Video (with background subtracted) of an animal partially immobilized in a small microfluidic chamber. The animal is initially quiescent but wakes upon illumination.

File Name: Supplementary Movie 4
Description: Microfluidic-induced sleep is reversible with strong mechanical stimulation. Two animals receive strong mechanical stimulation via a microfluidic push-down valve. The first animal is in the wake state, but behaviorally responds to stimulation. The second animal is in the sleep state and wakes upon stimulation.

File Name: Supplementary Movie 5
Description: Animals show a decreased response to weak mechanical stimuli during microfluidic-induced sleep. Two animals receive weak mechanical stimulation via a microfluidic push-down valve. The first animal is in the wake state, but still behaviorally responds to stimulation. The second animal is in the sleep state. Due to an increased arousal threshold, the animal does not respond.

File Name: Supplementary Movie 6
Description: Two representative examples of a brain and behavioral state transitions during microfluidic-induced sleep. Video shows single-plane, whole-brain epifluorescence of GCaMP6s imaging during a microfluidic-induced sleep transition. Top trace is animal behavioral activity. Middle trace shows the average ganglia fluorescence. Bottom trace shows the fluorescence of ten individual neurons.
