# Supplementary material for: A microfluidic-induced C. elegans sleep state
Source: Nat Commun. 2019 Nov 6;10:5035. doi: 10.1038/s41467-019-13008-5 (PMC6834590; doi:10.1038/s41467-019-13008-5)
Supplement: Supplementary file 10 — Reporting Summary [file 41467_2019_13008_MOESM10_ESM.pdf]

nature research

Corresponding author(s): Jacob T. Robinson

Last updated by author(s): Sep 16, 2019

Reporting Summary

Nature Research wishes to improve the reproducibility of the work that we publish. This form provides structure for consistency and transparency in reporting. For further information on Nature Research policies, see Authors & Referees and the Editorial Policy Checklist .

Statistics

For all statistical analyses, confirm that the following items are present in the figure legend, table legend, main text, or Methods section.

n/a	Confirmed	
☐	☒	The exact sample size (*n*) for each experimental group/condition, given as a discrete number and unit of measurement
☐	☒	A statement on whether measurements were taken from distinct samples or whether the same sample was measured repeatedly
☐	☒	The statistical test(s) used AND whether they are one- or two-sided<br>*Only common tests should be described solely by name; describe more complex techniques in the Methods section.*
☒	☐	A description of all covariates tested
☐	☒	A description of any assumptions or corrections, such as tests of normality and adjustment for multiple comparisons
☐	☒	A full description of the statistical parameters including central tendency (e.g. means) or other basic estimates (e.g. regression coefficient) AND variation (e.g. standard deviation) or associated estimates of uncertainty (e.g. confidence intervals)
☐	☒	For null hypothesis testing, the test statistic (e.g. *F*, *t*, *r*) with confidence intervals, effect sizes, degrees of freedom and *P* value noted<br>*Give P values as exact values whenever suitable.*
☒	☐	For Bayesian analysis, information on the choice of priors and Markov chain Monte Carlo settings
☒	☐	For hierarchical and complex designs, identification of the appropriate level for tests and full reporting of outcomes
☒	☐	Estimates of effect sizes (e.g. Cohen's *d*, Pearson's *r*), indicating how they were calculated

Our web collection on statistics for biologists contains articles on many of the points above.

Software and code

Policy information about availability of computer code

Data collection	Commercial imaging software was used for each camera. The Zyla camera (see Methods) uses Andor Solis. The Grasshopper and Basler cameras (see Methods) use commercial Point Grey and Basler interfaces, respectively.
Data analysis	Custom MATLAB 2018 scripts were used to analyze data except in the case of whole-brain imaging in behaving animals in which case we used the FIJI manual tracking plugin.

For manuscripts utilizing custom algorithms or software that are central to the research but not yet described in published literature, software must be made available to editors/reviewers. We strongly encourage code deposition in a community repository (e.g. GitHub). See the Nature Research guidelines for submitting code & software for further information.

Data

Policy information about availability of data

All manuscripts must include a data availability statement. This statement should provide the following information, where applicable:

- Accession codes, unique identifiers, or web links for publicly available datasets
- A list of figures that have associated raw data
- A description of any restrictions on data availability

A source data file have been provided with this submission and the manuscript includes a link to a BOX repository with all raw data. Upon request, we will also provide raw videos and analysis code.

Field-specific reporting

Please select the one below that is the best fit for your research. If you are not sure, read the appropriate sections before making your selection.

Life sciences study design

All studies must disclose on these points even when the disclosure is negative.

Sample size	Statistical analyses were not use to determine sample size before experiments. Rather, sample sizes were based on the scalability of experiments and are largely consistent with existing literature. This includes typically >20 animals for behavioral experiments and <10 animals for neural imaging experiments.
Data exclusions	Approximately 1% of animals were damaged by the microfluidic loading process, leading to severely impaired locomotion. We removed these animals from the analyses.
Replication	Rather than performing one experiment with a single population of animals, our experiments consist of multiple trials across multiple days with age-matched animals. Each individual trial typically includes imaging 1-15 animals simultaneously. These individual trials show the same trends as the full data set averaged across all trials.
Randomization	Controls were often interleaved with each experiment on the same imaging platform but were not randomized.
Blinding	No blinding was used in these experiments.

Reporting for specific materials, systems and methods

We require information from authors about some types of materials, experimental systems and methods used in many studies. Here, indicate whether each material, system or method listed is relevant to your study. If you are not sure if a list item applies to your research, read the appropriate section before selecting a response.

Materials & experimental systems

n/a	Involved in the study	
☒	☐	Antibodies
☒	☐	Eukaryotic cell lines
☒	☐	Palaeontology
☐	☒	Animals and other organisms
☒	☐	Human research participants
☒	☐	Clinical data

Methods

n/a	Involved in the study	
☒	☐	ChIP-seq
☒	☐	Flow cytometry
☒	☐	MRI-based neuroimaging

Animals and other organisms

Policy information about studies involving animals; ARRIVE guidelines recommended for reporting animal research

Laboratory animals	C. elegans. Specific mutant and transgenic strains are listed in the Methods.
Wild animals	No wild animals were used
Field-collected samples	Study did not involve field samples.
Ethics oversight	IACUC or other ethical oversight is not required for research involving invertebrates.

Note that full information on the approval of the study protocol must also be provided in the manuscript.
